# The Influence of Ginger Supplementation on Cycling Performance

**DOI:** 10.3390/sports14040126

**Published:** 2026-03-24

**Authors:** Jennifer A. Kurtz, Mabry Watson, Briana Robinson, Casey Edmondson, Laurel Wentz

**Affiliations:** 1 Department of Kinesiology, Appalachian State University, Boone, NC 28607, USA; 2 College of Health Sciences, The University of Memphis, Memphis, TN 38152, USA; bmrbnsn5@memphis.edu; 3 Department of Nutrition and Health Care Management, Appalachian State University, Boone, NC 28607, USAwentzlm@appstate.edu (L.W.)

**Keywords:** polyphenol, cycling performance, endurance, trained cyclists, oxygen consumption

## Abstract

Ginger supplementation is proposed as a natural ergogenic aid due to its anti-inflammatory and antioxidant properties, but its effects on endurance performance remain unclear. Methods: In this randomized, double-blind, placebo-controlled crossover trial, 30 trained cyclists (27 male, 3 females, aged 36 ± 11 yr) completed three visits: a baseline 75 km time trial, a post-supplementation time trial, and a second post-supplementation trial under the alternate condition. Participants consumed either ginger or placebo for four weeks. Statistics: Performance outcomes (i.e., 75 km time, VO_2_, power output, heart rate, and RPE) were analyzed using repeated-measures ANOVA, with repeated-measures ANCOVA to assess dietary and age influences (*p* < 0.05). Results: Energy and carbohydrate intake were consistent across trials and unrelated to performance. Protein intake per kg body mass predicted performance time in the placebo trial and average VO_2_ in the ginger trial; other macronutrients were not associated with outcomes. No significant differences were observed between ginger and placebo conditions for time to completion, VO_2_, power output, heart rate, or perceived muscle soreness. Conclusions: Four weeks of ginger supplementation does not improve prolonged cycling performance, recovery, or muscle soreness in trained cyclists when dietary intake is controlled. Future research should explore cellular mechanisms to determine whether ginger supplementation could influence performance or recovery in endurance athletes.

## 1. Introduction

Prolonged and intensive endurance exercise can markedly challenge physiological systems, contributing to fatigue, oxidative stress, inflammation, and delayed recovery, which ultimately impair subsequent performance. In competitive settings where reducing training volume and intensity is not practical, some athletes and coaches seek evidence-based nutritional approaches that support recovery and optimize performance outcomes. Dietary strategies that supply antioxidants and bioactive compounds have shown promise for attenuating exercise-related muscle damage and promoting faster recovery of muscle function, which can help maintain performance across repeated training sessions or competition days. Polyphenol-rich nutritional interventions, such as tart cherry, quercetin, and other flavonoid sources, have been investigated for their potential to improve endurance performance and recovery by reducing muscle soreness, oxidative stress, and inflammation after intense exercise [1,2,3,4,5]. Systematic reviews suggest that polyphenol supplementation may enhance aerobic endurance metrics (e.g., time to exhaustion, time-trial performance, distance covered to exhaustion) and recovery profiles, although evidence remains mixed and context-dependent [6].

Cycling performance is also strongly influenced by overall dietary intake, energy availability, and nutrient timing. In trained cyclists, carbohydrate availability plays a central role in supporting substrate utilization and sustaining high power output during prolonged efforts [7]. Adequate pre-race nutrition is therefore critical for performance and recovery. Previous work demonstrates substantial variability in dietary intake among professional cyclists, with reported energy intakes ranging from approximately 3000 kcal/day with 4–5 g·kg^−1^ carbohydrates [8] to upwards of 5000 kcal/day and 12 g·kg^−1^ carbohydrates during competitions [9]. Although evidence-based nutrition recommendations for cyclists exist and several studies have quantified the dietary habits of professional cyclists, research examining dietary intake and performance among amateur cyclists remains limited [10].

Ginger (*Zingiber officinale*) contains bioactive compounds with polyphenol-like properties and has a long history of use in Eastern and Ayurvedic medicine, making it a promising but underexplored nutritional strategy for endurance athletes. Its active constituents, gingerols, shogaols, and paradols, exert antioxidant and anti-inflammatory effects that may support endurance performance by attenuating exercise-induced oxidative stress and fatigue [11]. Most prior investigations have focused on ginger’s analgesic or antiemetic properties, or on muscle pain following eccentric or resistance exercise [12,13,14], with limited extension to endurance-based exercise models. A study in mice found that ginger extract increased muscle glycogen and improved running capacity [15]. However, no randomized controlled trials have examined ginger’s effects on endurance performance in humans or its role in improving performance to prolonged endurance exercise [16]. This gap highlights the need for targeted investigation in trained athletes.

Although evidence-based nutritional recommendations and performance outcomes have been extensively studied in professional endurance athletes, the relationship between habitual dietary intake and performance in high-level amateur cyclists remains less investigated. Moreover, despite growing interest in food-based strategies with potential ergogenic and recovery benefits, no human studies have examined the effects of ginger supplementation on endurance performance. This lack of evidence limits the translation of emerging nutritional strategies to applied practice in competitive amateur cycling and underscores the need for controlled investigations in this population. The purpose of this study was to examine the effects of four weeks of daily ginger supplementation on 75 km cycling time-trial performance, including time to completion, oxygen consumption (VO_2_), power output, and heart rate as primary outcomes. Secondary, exploratory analyses examined the influence of pre-exercise dietary intake and during-race nutrition on performance. We hypothesized that ginger supplementation would improve cycling performance and reduce perceived muscle soreness compared with placebo.

## 2. Materials and Methods

### 2.1. Study Design

**Figure 1:**
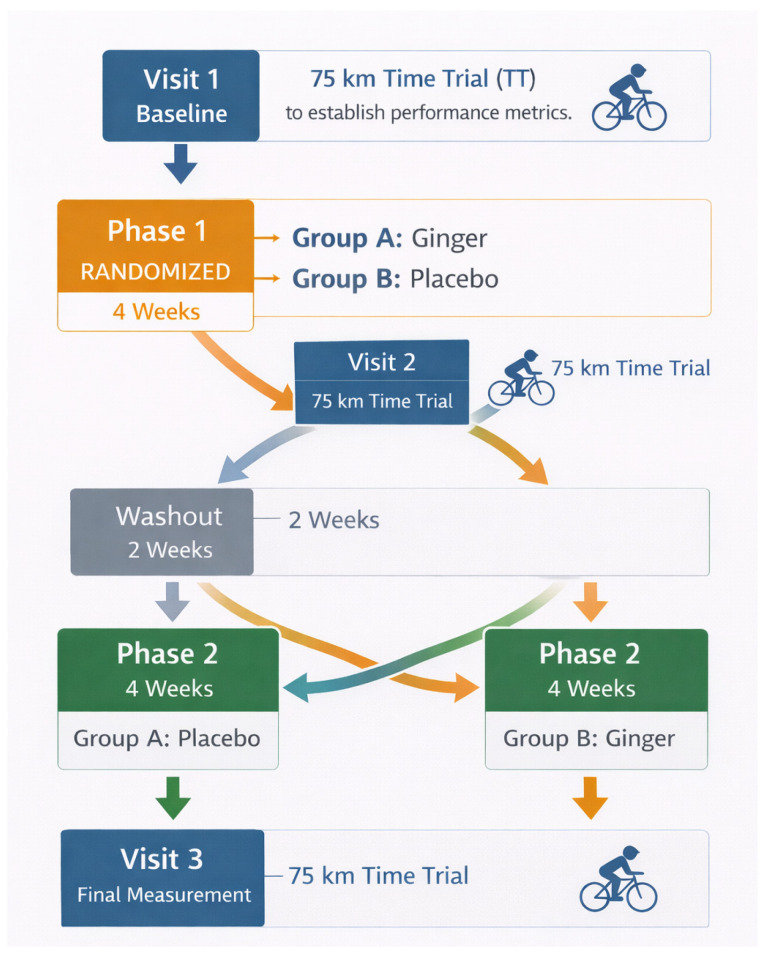
Experimental protocol and crossover study design. Trained endurance cyclists completed a 10-week randomized, double-blind, placebo-controlled crossover trial. Following baseline testing, Participants were assigned to either Group A or Group B, completing one condition first, followed by a 2-week washout period before crossing over to complete the alternate condition. Three identical 75 km simulated mountainous time trials (TT1–TT3) were completed. During each trial, VO_2_, heart rate, and RPE were recorded.

This study was a randomized, double-blind, placebo-controlled crossover trial examining the effects of four weeks of ginger supplementation on prolonged cycling performance in trained endurance athletes. Participants completed three 75 km laboratory-based cycling time trials separated by two 4-week supplementation phases (ginger or placebo) and a 2-week washout (Figure 1). The study protocol was approved by the Institutional Review Board at Appalachian State University (IRB #HS-24-170) and adhered to the Declaration of Helsinki.

### 2.2. Participants

Participants were men and women aged 18–60 years who were generally healthy, non-smokers, and free from chronic diseases (e.g., cardiovascular disease, diabetes, rheumatoid arthritis, or cancer, except non-melanoma skin cancer). Cyclists were required to have at least two years of experience, training a minimum of 3 sessions per week and 3–5 h per week, with cycling as their primary sport. Participants were classified as Tier 2 using a 6-tier framework for exercise and sports performance [17,18], defined as “trained, developed individuals who train with the purpose of competition.” This age range was selected to capture adult cyclists who are actively training while allowing for recruitment feasibility across a broad, real-world sample of trained endurance athletes. This design allowed examination of potential age-related variability in physiological and perceptual responses to endurance exercise and nutritional supplementation. Exclusion criteria included regular use of anti-inflammatory medications or high-dose supplements, chronic illness or injury limiting performance, tobacco use, excessive alcohol intake, “high risk” status per the ACSM health screening questionnaire, or allergy to ginger. Recruitment occurred via local cycling clubs, university networks, and social media.

### 2.3. Procedures

#### 2.3.1. Visit 1: Baseline Testing

Participants were instructed to avoid strenuous training intensity and volume for 48 h prior to testing and were instructed to consume a moderate carbohydrate diet (55–65% of daily intake). On arrival, informed consent, medical screening, 24 h dietary recall (NIH ASA24), sleep quality assessment (shortened PSQI), anthropometrics, body mass, body composition (DEXA), and hydration status (urine specific gravity) were assessed. Pre-trial perceived muscle soreness (0–10 scale) was recorded.

Participants then completed a 75 km simulated mountainous time trial on a Wahoo Kickr Core Smart Trainer (Wahoo Fitness, Atlanta, GA, USA) integrated with Zwift software (Zwift Inc., Long Beach, CA, USA; https://www.zwift.com). Gas exchange data (VO_2_, respiratory exchange ratio [RER]) were measured using a calibrated COSMED K5 Portable Metabolic Analyzer (COSMED, Rome, Italy; OMNIA software (version 2.5.1) device firmware version 2.1.). Heart rate was monitored continuously (Polar H10), and RPE was recorded every 10 km using the Borg 6–20 scale. Participants self-paced to maximize completion speed and consumed fluids/foods ad libitum. Post-trial muscle soreness was reassessed. Participants were then provided a 4-week supply of either ginger or placebo supplements.

#### 2.3.2. Supplementation

Participants consumed either ginger or placebo supplements for four weeks in a randomized order. Each daily dose consists of two 60 mL shots (morning and evening). The ginger supplement (Pure Green Immunity Shot, Pure Green Franchise LLC, New York, NY, USA) contained 53 g juiced ginger in lemon juice, providing 25 kcal and 7 g carbohydrate per daily dose (two 60 mL shots). Gingerols (6-, 8-, and 10-gingerol) were quantified by High-Performance Liquid Chromatography with Electrochemical Detection. The placebo was ginger ale matched for carbohydrate and energy content, containing <2% ginger extract. Compliance and gastrointestinal tolerance were monitored weekly via a 16-item questionnaire (Qualtrics, Provo, UT, USA), including prompts for supplement dosing diaries. Participants maintained habitual cycling training throughout, tracked via Strava. Questionnaire items and symptom domains were adapted from validated GI distress assessment methods and prior supplementation research, including abdominal pressure and distension, belching, difficulty with gas evacuation, flatulence, nausea, heartburn, and bowel movement abnormalities [19].

#### 2.3.3. Visit 2: Post-Supplementation (First Condition)

After four weeks of supplementation, participants repeated the time-trial procedures, maintaining the same diet and training conditions as baseline.

#### 2.3.4. Washout Period

Following the first intervention, participants completed a two-week washout period before beginning the alternate supplementation phase. This duration is consistent with prior sports nutrition crossover trials examining dietary supplements and polyphenol-rich interventions in endurance athletes and was selected to minimize potential carryover effects while maintaining practical feasibility for longitudinal testing [20,21,22]. Our crossover design implementation and selected washout period allowed residual effects to subside and are considered standard practice for reversible outcomes in nutritional interventions. Participants abstained from all other sport supplementation while recording training load and dietary habits. After two weeks, participants consumed the alternate supplement condition.

#### 2.3.5. Visit 3: Post-Supplementation (Second Condition)

Participants completed the alternate supplementation for four weeks, then repeated all assessments and time-trial procedures as in Visit 2.

### 2.4. Statistical Analysis

An a priori power analysis was conducted using *GPower 3.1, informed by effect sizes reported in previous time-trial performance research. For example, protocols involving cycling time trials have demonstrated appreciable differences in completion time and power output between conditions [23,24], and reliability data from 5 km TTs indicate measurable sensitivity in performance time as an outcome [25]. The sample size for this study was determined as part of a larger trial designed to assess recovery and immune function. An a priori power analysis (G*Power 3.1) indicated that 19 participants would provide 80% statistical power (α = 0.05) to detect a within-subject difference with a large effect size (Cohen’s *d* = 0.7) for the primary immune outcome. In the present manuscript, the cycling performance outcomes were considered exploratory and therefore were not used to determine the required sample size. Statistical analysis was computed using the generalized linear model (GLM) repeated-measures ANOVA module in SPSS (version 31, IBM Corp., Armonk, NY, USA). Data were analyzed using generalized linear models. A two-way repeated-measures ANOVA was used to examine the main effects of the 2 treatments (ginger vs. placebo) across 9 time points (0, 5, 15, 25, 35, 45, 55, 65, and 75 km), as well as the treatment × time interaction. For heart rate, an additional 38 km time point was included in the analysis (2 × 10). A 2 × 2 between-subjects ANOVA was conducted to examine whether intervention order affected time to completion across the ginger and placebo conditions. A one-way repeated-measures ANOVA was conducted to determine if nutrients differed between each time trial. A repeated-measures ANCOVA was conducted to examine whether dietary intake predicted cycling performance outcomes across the three trials. Gastrointestinal distress responses were analyzed descriptively by condition, and differences between ginger and placebo were evaluated using McNemar’s tests for paired categorical data.

To assess potential carryover effects, order effects (placebo-first vs. ginger-first) were evaluated using one-way analyses of variance for time-trial completion time, average VO_2_, power output, heart rate, and ratings of perceived exertion to support adequate washout period. ANCOVAs were performed for performance outcome variables (i.e., time to completion for time-trial completion time, average VO_2_, power output, heart rate, and ratings of perceived exertion) with age as a covariate and treatment order included where possible. This allowed us to account for potential differences in physiological response across the age range.

Normality was verified with Shapiro–Wilk tests, and Mauchly’s test of sphericity was conducted; Greenhouse–Geisser corrections were applied when the assumption of sphericity was violated. When significant main effects or interactions were detected (*p* ≤ 0.05), follow-up pairwise comparisons were performed using Bonferroni-adjusted paired *t*-tests. Effect sizes (partial eta squared for ANOVA, Cohen’s *d* for pairwise comparisons) were calculated to quantify the magnitude of effects. Data are reported as mean ± standard deviation (SD), and 95% confidence intervals are provided where appropriate.

## 3. Results

### 3.1. Study Participant Demographics and Anthropometrics

Thirty trained cyclists (27 males, 3 females; age 36 ± 11 yr) participated in the study, providing a broad adult age range that enhances the generalizability of findings across different age groups. Participants had a mean height of 177.7 ± 9.0 cm, body mass of 78.4 ± 12.9 kg, fat-free mass of 63.6 ± 10.2 kg, fat mass of 14.8 ± 3.7 kg, body fat percentage of 18.8 ± 2.9%, reported average weekly cycling volume of 89.6 ± 48.0 miles, and 7.43 ± 0.53 reported hours of sleep per night. Participants maintained their habitual cycling training throughout the study, with no significant differences in training volume or intensity between experimental conditions (*p* > 0.05).

Participant anthropometrics, body composition, weekly cycling volume, and habitual sleep duration were not statistically significant predictors of time-trial completion and were not retained as covariates in either the ginger or placebo condition (all *p* > 0.05). All subjects started cycling hydrated according to their urine specific gravity (USG < 1.020). Mean and standard deviation for USG were as follows: baseline TT 1.007 ± 0.007; ginger TT 1.009 ± 0.007; placebo TT 1.007 ± 0.006.

**Figure 2:**
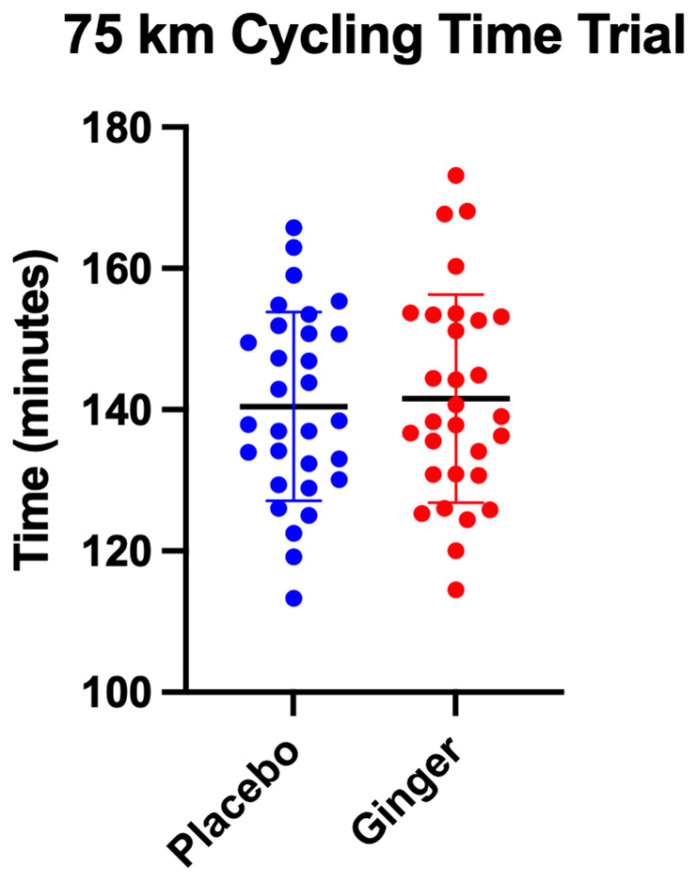
Comparisons of average time-trial completion across conditions at each time point during the 75 km time trial. Data are presented as mean ± standard deviation. Plotted dots show individual subject data.

Order effects (placebo-first vs. ginger-first) were evaluated to assess potential carryover in the crossover design. No significant order effects were observed for time-trial completion time, average VO_2_, power output, heart rate, or ratings of perceived exertion (all *p* > 0.05), indicating that the sequence of supplementation did not influence physiological or performance outcomes. There was no significant effect of intervention order on time-trial completion (F(1, 28) = 1.71, *p* = 0.20, η^2^_p_ = 0.06, 95% CI [0.00, 0.23]). There was also no significant effect of condition (ginger vs. placebo; F(1, 28) = 0.49, *p* = 0.49, η^2^_p_ = 0.02, 95% CI [0.00, 0.16]) and no condition × order interaction (F(1, 28) = 0.42, *p* = 0.52, η^2^_p_ = 0.02, 95% CI [0.00, 0.15]). Average completion times were similar between supplementation sequences, with 141.3 ± 2.7 min for the ginger-first group and 139.9 ± 2.4 min for the placebo-first group (Figure 2). Time to completion was not significantly affected by age or treatment order in the placebo or ginger condition (*p* > 0.05), indicating that completion times were generally consistent across participants after adjusting for these factors.

**Figure 3:**
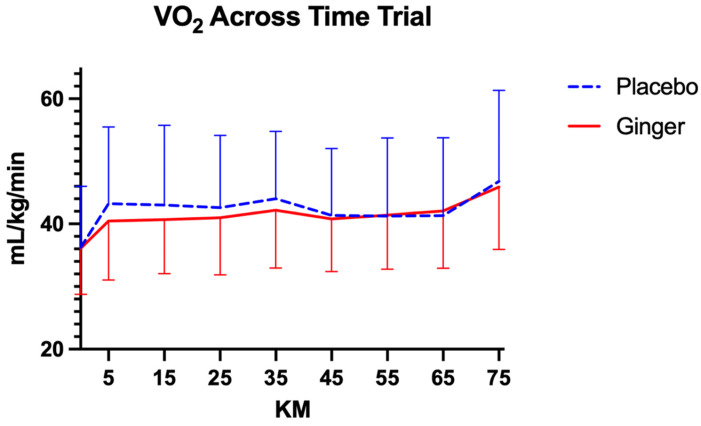
Comparisons of average VO_2_ across conditions at each time point during the 75 km time trial. Data are presented as mean ± standard deviation.

Average VO_2_ during the time trial was similar between supplementation conditions. Participants exhibited an average VO_2_ of 42.07 ± 3.24 mL·kg^−1^·min^−1^ across repeated measurements following ginger supplementation and 42.45 ± 2.83 mL·kg^−1^·min^−1^ following placebo (Figure 3). VO_2_ changed significantly over time in both conditions, with a within-subjects effect of time for placebo (F(9, 252) = 9.44, *p* < 0.00, η^2^_p_ = 0.25, 95% CI [0.15, 0.33]) and ginger (F(8, 208) = 17.62, *p* < 0.00, η^2^_p_ = 0.40, 95% CI [0.30, 0.47]). The time × intervention order interactions were not significant for placebo (F(9, 252) = 0.81, *p* = 0.61, η^2^_p_ = 0.02, 95% CI [0.00, 0.05]) or ginger (F(8, 208) = 0.83, *p* = 0.58, η^2^_p_ = 0.03, 95% CI [0.00, 0.07]), indicating that changes in VO_2_ over time were similar regardless of supplementation sequence. For the placebo condition, age did not significantly influence average VO_2_ (F(1, 15) = 1.01, *p* = 0.33; R^2^ = 0.06). In contrast, for the ginger condition, age was a significant covariate (F(1, 11) = 8.33, *p* = 0.02; R^2^ = 0.43), indicating that older participants had different VO_2_ responses to ginger supplementation. Treatment order was not significant in either condition.

**Figure 4:**
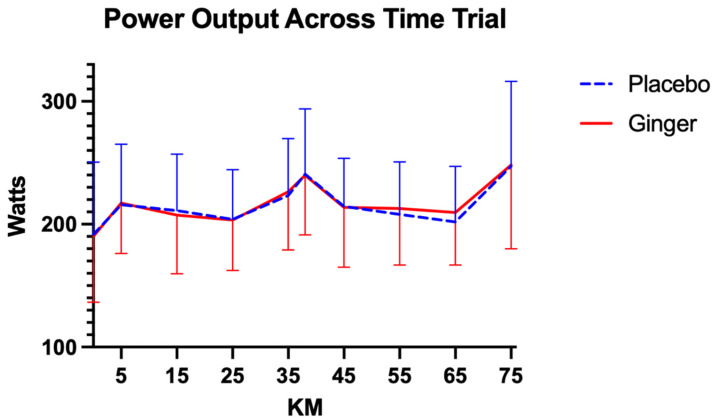
Comparisons of average power across conditions at each time point during the 75 km time trial. Data are presented as mean ± standard deviation.

Average power output during the time trial was similar between conditions. Participants produced a mean power of 218.63 ± 16.82 W following ginger supplementation and 217.17 ± 12.77 W following placebo (Figure 4). For both conditions, there was a significant within-subjects effect of time on average power output, indicating changes across the trial (placebo: F(8, 224) = 7.71, *p* < 0.00, η^2^_p_ = 0.22, 95% CI [0.12, 0.30]; ginger: F(8, 216) = 9.11, *p* < 0.00, η^2^_p_ = 0.25, 95% CI [0.16, 0.33]). The time × intervention order interaction was not significant in either condition (placebo: F(8, 224) = 0.73, *p* = 0.67, η^2^_p_ = 0.03, 95% CI [0.00, 0.07]; ginger: F(8, 216) = 1.15, *p* = 0.33, η^2^_p_ = 0.04, 95% CI [0.00, 0.09]), and between-subjects analyses showed no significant effect of intervention order on average power output (placebo: F(1, 28) = 1.62, *p* = 0.21, η^2^_p_ = 0.06, 95% CI [0.00, 0.22]; ginger: F(1, 27) = 1.50, *p* = 0.23, η^2^_p_ = 0.05, 95% CI [0.00, 0.21]). Average power was not significantly affected by age or treatment order in either the placebo or ginger conditions (*p* > 0.05), indicating that responses were generally consistent across participants.

**Figure 5:**
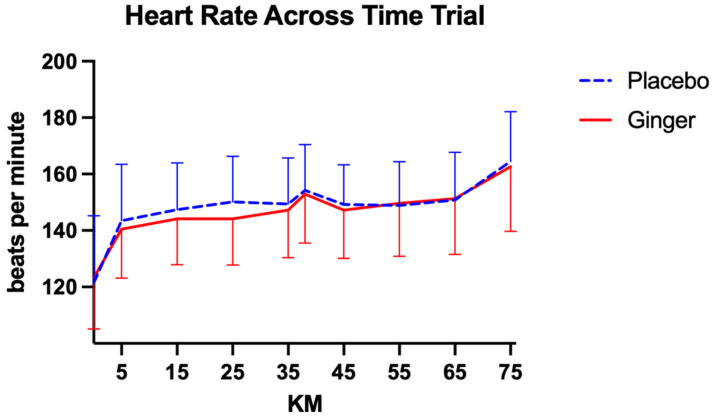
Comparisons of average HR across conditions at each time point during the 75 km time trial. Data are presented as mean ± standard deviation.

Average heart rate during the time trial was similar across conditions. Participants exhibited a mean HR of 146.55 ± 11.27 bpm following ginger supplementation and 147.97 ± 8.70 bpm following placebo (Figure 5). For both conditions, there was a significant within-subjects effect of time on heart rate, indicating increases across the trial (ginger: F(9, 243) = 27.71, *p* < 0.00, η^2^_p_ = 0.51, 95% CI [0.44, 0.57]; placebo: F(9, 207) = 27.60, *p* < 0.00, η^2^_p_ = 0.55, 95% CI [0.48, 0.61]). The time × intervention order interaction was not significant in either condition (ginger: F(9, 243) = 0.21, *p* = 0.99, η^2^_p_ = 0.01, 95% CI [0.00, 0.03]; placebo: F(9, 207) = 0.94, *p* = 0.50, η^2^_p_ = 0.04, 95% CI [0.00, 0.09]). Average heart rate was significantly associated with age in both conditions, with older participants exhibiting higher HR (placebo: F(1, 27) = 6.02, *p* = 0.02, η^2^_p_ = 0.18, 95% CI [0.01, 0.43]; ginger: F(1, 27) = 6.74, *p* = 0.02, η^2^_p_ = 0.20, 95% CI [0.02, 0.45]). Treatment order did not significantly influence HR in the placebo condition or in the ginger condition (*p* > 0.05).

**Figure 6:**
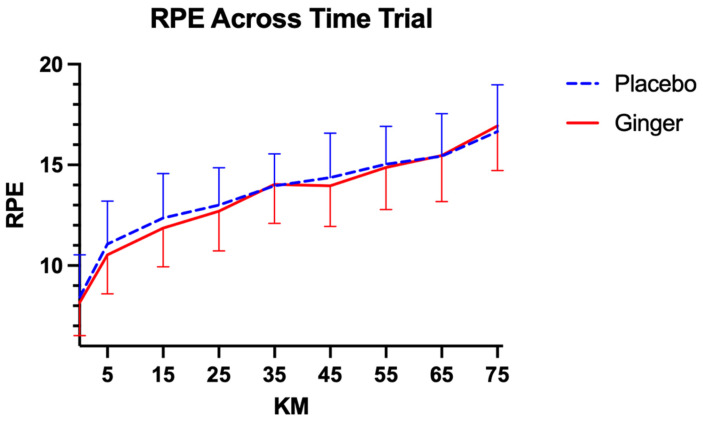
Comparisons of average RPE across conditions at each time point during the 75 km time trial. RPE was measured using the 6–20 Borg RPE scale, where 6 indicates no exertion and 20 represents maximal exertion. Data are presented as mean ± standard deviation.

RPE increased from the start to the end of exercise in both conditions, from 8.44 ± 0.40 at 0 km to 16.71 ± 0.44 at 75 km for placebo, and from 8.22 ± 0.30 to 16.99 ± 0.41 for ginger supplementation (Figure 6). RPE increased significantly over time in both conditions. For the placebo condition, there was a significant within-subjects effect of time (F(8, 216) = 72.40, *p* < 0.00, η^2^_p_ = 0.73, 95% CI [0.67, 0.78]), with no significant time × intervention order interaction (F(8, 216) = 0.64, *p* = 0.74, η^2^_p_ = 0.02, 95% CI [0.00, 0.05]); average RPE increased from 8.44 ± 0.40 at 0 km to 16.71 ± 0.44 at 75 km. Similarly, for the ginger condition, there was a significant effect of time (F(8, 224) = 122.35, *p* < 0.00, η^2^_p_ = 0.81, 95% CI [0.77, 0.85]), with no time × intervention order interaction (F(8, 224) = 0.49, *p* = 0.86, η^2^_p_ = 0.02, 95% CI [0.00, 0.05]); average RPE increased from 8.22 ± 0.30 at 0 km to 16.99 ± 0.41 at 75 km. Across both conditions, older participants reported slightly higher average RPE (placebo: F(1, 26) = 4.57, *p* = 0.04, η^2^_p_ = 0.15, 95% CI [0.00, 0.39]; ginger: F(1, 27) = 5.86, *p* = 0.02, η^2^_p_ = 0.18, 95% CI [0.01, 0.42]).

In the ginger condition, there was a significant effect of intervention order (F(1, 28) = 4.21, *p* = 0.05, partial η^2^ = 0.13, 95% CI [0.00, 0.36]), with participants completing ginger first showing higher absolute instantaneous jump power performance (PP = 1671 ± 209 W) than those completing placebo first (PP = 1403 ± 71 W). In the placebo condition, there was no significant effect of intervention order (PP = 1384 ± 73 W vs. 1402 ± 73 W). In contrast, the placebo condition showed no significant effect of intervention order (F(1, 27) = 2.55, *p* = 0.12, partial η^2^ = 0.09, 95% CI [0.00, 0.30]), with similar performance between order groups (ginger, placebo: 1384 ± 73 W; placebo, ginger: 1402 ± 73 W).

Cyclists maintained a consistent diet in the 24 h preceding each time trial, consuming macronutrients that met general cycling recommendations. For example, mean carbohydrate intake exceeded 5 g/kg, and mean protein intake ranged from 1.6 to 1.9 g/kg of body mass (Table 1). Macronutrients, notably omega-3 fatty acids and fiber, met recommendations for endurance athletes.

Cyclists’ micronutrient intake did not differ between cycling trials (Table 2). Vitamin C intake was higher prior to the baseline ride (F(2, 56) = 4.11, *p* = 0.02, η^2^_p_ = 0.13, 95% CI [0.01, 0.29]) but was consistent for ginger and placebo interventions.

Cyclists ate ad libitum for each time trial, instructed to follow their typical race behavior (Table 3). They were asked to repeat the same fueling strategies for each time trial, and data collected show no significant differences across time trials.

#### 3.1.1. Diet and Performance

Neither energy intake nor carbohydrate intake predicted time to completion across trials. However, protein intake per kg predicted performance time in the placebo trial (F(1, 25) = 5.66, *p* = 0.03, η^2^_p_ = 0.19, 95% CI [0.02, 0.42]), indicating a moderate effect, although the effect was not significant for baseline or ginger rides. On the other hand, protein intake per kg significantly predicted average VO_2_ for the ginger time trial (F(1, 25) = 5.04, *p* = 0.03, η^2^_p_ = 0.17, 95% CI [0.01, 0.39]), while protein was not significant for the baseline or placebo ride. No other macronutrient consumed in the 24 h period preceding the time trial or during the time trial predicted time, average VO_2_, heart rate, or power output.

#### 3.1.2. Gastrointestinal Distress

Gastrointestinal distress symptoms were negligible across conditions, with few symptoms reported in responses for both placebo (≥27 of 30 responses) and ginger (≥26 of 30 responses) supplementation conditions. Across all survey items, including bloating, abdominal pain, nausea, heartburn, and abnormal bowel movements, responses were negative for both conditions, with only isolated reports of flatulence, belching, or feelings of fullness (≤2 participants per condition). Overall, both ginger and placebo were well tolerated and did not elicit clinically meaningful gastrointestinal discomfort.

## 4. Discussion

### 4.1. Performance Outcomes

This study is the first to examine the effects of a 4-week ginger supplementation protocol on prolonged cycling performance in trained cyclists. Overall, ginger supplementation did not significantly alter power output, VO_2_, heart rate, or RPE compared with placebo. Although no significant treatment × time interactions were observed, VO_2_ values were generally slightly higher in the placebo condition. These small, non-significant differences should be interpreted cautiously given the study was not powered to detect small performance-related changes, and, together with trivial effect sizes and confidence intervals, suggest that ginger supplementation likely had minimal impact on oxygen cost during prolonged cycling [12]. These findings suggest that ginger supplementation alone is unlikely to meaningfully modify oxygen demand, cardiovascular responses, or perceptual effort during prolonged cycling in trained athletes.

Our findings align with prior research on polyphenol or antioxidant supplementation in trained endurance athletes, which frequently reports inconsistent effects on aerobic metabolism and performance outcomes [26]. For instance, acute or short-term ginger supplementation (e.g., 2 g in young adults) has not been shown to significantly alter VO_2_, heart rate, work rate, or recovery oxygen kinetics during moderate-intensity cycling [12].

Similarly, controlled trials of polyphenol supplementation in trained cyclists or other endurance athletes often demonstrate modest improvements in recovery markers or muscle damage without corresponding changes in performance or physiological responses [27,28,29]. Systematic reviews of ginger’s ergogenic properties conclude that while ginger may modestly reduce muscle pain or anti-inflammatory and anti-oxidative effects, these mechanisms do not reliably translate to enhancements in oxygen utilization, power output, cardiovascular responses, or perceptual effort during exercise [6,27,30,31,32,33,34].

Collectively, the absence of meaningful differences in VO_2_, power output, heart rate, and perceived exertion suggests that ginger supplementation did not substantially alter the physiological demands of prolonged cycling in trained athletes. This aligns with the broader polyphenol literature, which frequently reports small or variable effects on performance markers in well-trained populations [35]. As athletes approach their physiological ceiling, improvements in VO_2_max and related cardiorespiratory variables become progressively smaller, reflecting the principle of diminishing returns in endurance training adaptations. Consequently, nutritional interventions such as ginger supplementation may exert only subtle effects on acute physiological responses. Overall, four weeks of ginger supplementation did not meaningfully modify oxygen consumption, cardiovascular responses, power output, or perceived exertion, indicating that its antioxidant and anti-inflammatory properties may not translate into measurable changes in acute performance under the conditions tested.

### 4.2. Age Effects on Performance Variables

In the present study, the influence of age did not significantly influence time to completion or average power, suggesting that cycling performance outcomes were largely consistent across the participant age range. This finding aligns with prior research indicating that well-trained endurance athletes can maintain performance across a broad adult age span, particularly in events lasting less than ~2–3 h [36,37] (Tanaka & Seals, 2008; Lepers & Stapley, 2016). These results support the use of a heterogeneous age sample in endurance trials without introducing major confounding for performance outcomes.

However, physiological and perceptual responses were affected by age. In the placebo condition, age did not significantly influence average VO_2_ (*p* = 0.33), whereas in the ginger condition, age was a significant covariate (*p* = 0.02), indicating that older participants responded differently in terms of oxygen uptake to ginger supplementation. These results are consistent with prior studies demonstrating age-related declines in maximal oxygen consumption and exercise economy, even in trained populations [36,38]. Such changes may reflect diminished cardiac output, altered muscle oxidative capacity, and slower oxygen kinetics in older adults [39,40]. Similarly, age significantly influenced average heart rate and perceived exertion in both conditions, with older participants exhibiting higher HR and slightly elevated RPE. These findings are consistent with well-established age-associated cardiovascular and perceptual changes, including reduced maximal heart rate, diminished stroke volume, and greater relative cardiovascular strain at a given workload [36]. Overall, these results suggest that while age does not substantially affect gross performance metrics such as time to completion or power output, it does influence cardiovascular and perceptual responses, as well as the VO_2_ response to nutritional interventions like ginger. This has implications for interpreting endurance performance in master athletes and highlights the importance of considering age when assessing physiological responses to supplements or interventions.

### 4.3. Hydration

No significant difference in hydration markers was observed between conditions in our current study. Polyphenol supplementation has not been widely associated with direct effects on hydration status in the exercise physiology literature. Systematic reviews of polyphenols primarily highlight impacts on oxidative stress, inflammation, and some performance indicators, but effects on electrolyte balance, total body water, or hydration biomarkers are rarely reported and, when assessed, are typically non-significant [6]. This absence of hydration effects in the current results therefore mirrors the broader literature and suggests that, if polyphenols influence exercise performance, mechanisms other than fluid balance such as modulation of oxidative stress, inflammation, or endothelial function may be the most relevant areas for future mechanistic investigation. Consequently, future studies should prioritize exploring these cellular and systemic pathways to better understand how polyphenol supplementation might confer performance or recovery benefits, particularly in trained endurance athletes.

### 4.4. Nutritional Effects

Per instruction, cyclists maintained a similar diet across the three time trials, which likely explains why diet was not a strong predictor of performance outcomes. Vitamin C intake was higher prior the baseline time trial, although this had no effect on performance following ginger or placebo supplements as vitamin C and all other nutrients were consistent prior to the intervention rides. Research on dietary intake in amateur cyclists remains sparse compared with the detailed nutritional profiling available for professional cyclists, and our study is one of the first to publish quantitative dietary data on highly trained amateur cyclists. Previous research surveyed amateur cyclists using questionnaires, finding that most athletes consumed four meals per day and reported paying attention to their diet [41]. However, fruit and vegetable intake was below recommendations. Research on professional cyclists has been mixed, with some data showing cyclists meeting energy and macronutrient recommendations for endurance athletes, while other data show carbohydrate consumption below their fueling needs [8,9]. Our data support that amateur cyclists consume 5 g/kg carbohydrates, meeting the lower end of recommendations for sport [10]. However, carbohydrate intake during cycling was less than 30 g/h, falling below recommendations for endurance activity exceeding one hour.

Protein intake was slightly higher prior to the placebo trial (1.9 g/kg vs. 1.6 g/kg) and was a significant predictor of time to completion for the placebo time trial and VO_2_ for the ginger time trial. The majority of research shows that protein alone does not acutely enhance performance, though adding protein to carbohydrates may improve outcomes likely due to the additional calories rather than the protein itself [42]. However, given the lower range of carbohydrate intake in our cyclists, the additional protein intake may have compensated for any deficits in overall energy. Overall, our cyclists demonstrated adequate intake of key nutrients, including fiber, omega-3 fatty acids, and essential vitamins and minerals, suggesting careful dietary practices to support performance, similar to previously surveyed amateur cyclists [41]. These findings highlight that, while nutrition was generally sufficient, individual variation in protein and energy intake may still influence performance outcomes, representing a potential limitation of the study.

One important limitation of the current study is the relatively small sample size. While our performance markers did not reach statistical significance, the present study was not powered to detect small changes in performance or metabolic outcomes [43]. Similarly, many previous studies had smaller participant pools, which may contribute to the lack of statistically significant performance findings [32,44,45]. Further, previous performance studies have analyzed outcomes using a between–within repeated-measures ANOVA (RM-ANOVA). However, this approach typically requires effect sizes such as partial eta squared (η^2^) or Cohen’s *f*, which can be less intuitive than Cohen’s *d* commonly used in pre–post designs. As a result, conducting a power analysis for RM-ANOVA can be challenging for researchers investigating performance outcomes [23]. It is more likely that ginger enhances muscle recovery by modulating immune function rather than improving time-trial performance or power [46]. Another limitation is that biomarkers of oxidative stress, antioxidant capacity, or related mechanistic pathways were not measured in the present analysis. Because ginger’s proposed ergogenic effects are largely attributed to its antioxidant and anti-inflammatory properties, the absence of these measurements limits our ability to determine whether the lack of performance effects reflects supplement inefficacy or a mismatch between the physiological perturbation induced by the exercise protocol and the mechanisms through which ginger may exert benefits. Additional limitations should be considered when interpreting these findings. The use of trained endurance athletes may have limited the observable effects of supplementation, as this population typically exhibits reduced trainability of VO_2_ and performance metrics due to physiological ceiling effects, which can obscure potential benefits of nutritional interventions. Furthermore, variability in participant characteristics, including fitness level, sex, age, and prior supplementation history, may have contributed to interindividual responses and increased outcome variability. Although age did not significantly affect time-trial performance or power in this sample, the wide age range (18–55 yr) may have influenced physiological responses such as VO_2_, heart rate, and perceived exertion, potentially increasing outcome variability. Future studies should consider stratifying or controlling for age to better understand age-specific responses to supplementation. The duration of supplementation may also be a limiting factor, as a four-week intervention may be acute and insufficient to elicit measurable performance adaptations, and differences between acute and chronic supplementation strategies as well as timing relative to exercise could influence efficacy. Finally, the selected outcome measures (i.e., VO_2_, power, heart rate, and perceived muscle soreness) may lack the sensitivity required to detect subtle physiological or perceived muscle soreness recovery. In this context, biomarkers of inflammation, oxidative stress, or muscle damage may provide more mechanistic insight into the potential effects of ginger supplementation and should be prioritized in future investigations. An additional methodological limitation is that baseline physiological measurements were not repeated following the washout period prior to the second intervention phase. Although statistical analyses indicated no order effects across performance and cardiorespiratory outcomes, physiological equivalence between intervention phases was assumed rather than directly verified. Biomarkers related to oxidative stress, inflammation, or muscle damage were not measured at the start of each supplementation phase. Because these pathways were proposed as potential mechanisms through which ginger could influence exercise responses, future studies should include repeated baseline assessments of inflammatory and oxidative stress markers to better characterize mechanistic effects of supplementation. A further limitation of this study is that the trial was not prospectively registered in a publicly accessible clinical trial registry. Although the study received institutional ethical approval prior to initiation, prospective trial registration is now considered best practice for enhancing transparency and reproducibility in clinical research.

## 5. Conclusions

This randomized, double-blind crossover trial examined the effects of four weeks of ginger supplementation on prolonged cycling performance in trained endurance athletes. Ginger supplementation did not significantly affect time-trial completion time, oxygen consumption, power output, heart rate, perceived exertion, or hydration status compared with placebo. These findings suggest that ginger supplementation alone does not provide a meaningful ergogenic benefit for prolonged cycling performance in trained athletes. Future studies with larger samples, longer supplementation periods, and mechanistic biomarkers of inflammation, oxidative stress, and muscle damage are needed to determine whether ginger may indirectly support endurance performance through recovery processes.

## Figures and Tables

**Table 1:** Participants’ 24 h macronutrient dietary intake prior to each 75 km time trial (TT).

Nutrient	Baseline TT	Ginger TT	Placebo TT	Significance (*p*-Value)
Energy (Kcal)	3203 ± 961	3206 ± 978	3377 ± 947	0.50
Kcal/kg BM	41.8 ± 12.9	42.4 ± 15.5	44.8 ± 15.2	0.41
Carbohydrates (g)	389 ± 137	383 ± 134	383 ± 146	0.81
Carbohydrate g/kg BM	5.1 ± 1.9	5.1 ± 2.1	5.1 ± 2.2	0.84
Protein (g)	127 ± 56	124 ± 46	145 ± 50	0.10
Protein g/kg BM	1.6 ± 0.7	1.6 ± 0.6	1.9 ± 0.8	0.09
Fat (g)	126 ± 47	128 ± 54	139 ± 59	0.35
Saturated Fat (g)	41 ± 18	42 ± 23	46 ± 22	0.58
Cholesterol (mg)	439 ± 389	370 ± 323	401 ± 274	0.56
Omega-3 Fatty Acid (mg)	1091 ± 2170	1291 ± 2295	2013 ± 4091	0.39
Omega-6 Fatty Acid (mg)	5996 ± 5485	6814 ± 6598	6588 ± 8146	0.87
Fiber (g)	35 ± 14	32 ± 15	32 ± 15	0.267

Data are presented as mean ± standard deviation. There were no significant differences between macronutrients consumed prior to each time trial.

**Table 2:** Participants’ 24 h micronutrient dietary intake prior to each 75 km time trial (TT).

Nutrient	Baseline TT	Ginger TT	Placebo TT	Significance (*p*-Value)
Vitamin A (mg)	1156 ± 654	1063 ± 600	1363 ± 1359	0.32
Thiamin (mg)	3.0 ± 1.2	3.1 ± 1.5	2.9 ± 1.3	0.84
Riboflavin (mg)	3.5 ± 1.4	3.3 ± 1.4	3.5 ± 1.3	0.63
Niacin (mg)	40 ± 19	42 ± 20	45 ± 19	0.53
Vitamin B6 (mg)	3.5 ± 1.7	3.3 ± 1.3	3.5 ± 1.7	0.68
Folate (μg)	897 ± 479	902 ± 465	850 ± 512	0.67
Vitamin B12 (μg)	6.2 ± 4.3	7.1 ± 4.2	8.2 ± 6.2	0.11
Vitamin C (mg)	173 ± 126 *	127 ± 106	118 ± 101	0.00 *
Vitamin D (IU)	269 ± 198	365 ± 388	463 ± 522	0.11
Calcium (mg)	1498 ± 734	1510 ± 629	1785 ± 735	0.06
Iron (mg)	23 ± 10	23 ± 8.7	22 ± 9	0.76
Zinc (mg)	17 ± 7	17 ± 6	18 ± 6	0.50
Phosphorus (mg)	2133 ± 789	2155 ± 744	2398 ± 768	0.14
Magnesium (mg)	570 ± 231	581 ± 258	552 ± 191	0.79
Potassium (mg)	3994 ± 1435	4058 ± 1729	4257 ± 1436	0.48
Sodium (mg)	4547 ± 1577	4802 ± 2109	4994 ± 1695	0.42

Data are presented as mean ± standard deviation. * Vitamin C intake before baseline TT was significantly higher than ginger TT (*p* = 0.05) and placebo TT (*p* = 0.01), but there was no difference in vitamin C intake between ginger and placebo TT (*p* = 0.62).

**Table 3:** Dietary intake during each 75 km time trial (TT).

Nutrient	Baseline TT	Ginger TT	Placebo TT	STATS (*p*-Value)
Energy (Kcal)	272 ± 201	303 ± 302	261 ± 349	0.53
Kcal/kg BM	3.8 ± 3.1	4.1 ± 4.3	3.6 ± 4.9	0.57
Carbohydrates (g)	56 ± 41	62 ± 55	53 ± 62	0.52
Carbohydrate g/kg BM	0.8 ± 0.6	0.9 ± 0.8	0.7 ± 0.9	0.55

Data are presented as mean ± standard deviation. There were no significant differences between kilocalories (Kcals) and carbohydrates consumed during each time trial.

## Data Availability

The data that support the findings of this study are not publicly available due to privacy and ethical restrictions protecting participant confidentiality. Access to deidentified data may be considered on a case-by-case basis upon reasonable request to the corresponding author, in accordance with institutional and ethical guidelines.

## References

[1] Connolly D., McHugh M., Padilla-Zakour O. (2006). Efficacy of a tart cherry juice blend in preventing the symptoms of muscle damage. Br. J. Sports Med..

[2] Mashhadi N.S., Ghiasvand R., Askari G., Hariri M., Darvishi L., Mofid M.R. (2013). Anti-oxidative and anti-inflammatory effects of ginger in health and physical activity: Review of current evidence. Int. J. Prev. Med..

[3] McAnulty L.S., Miller L.E., Hosick P.A., Utter A.C., Quindry J.C., McAnulty S.R. (2013). Effect of resveratrol and quercetin supplementation on redox status and inflammation after exercise. Appl. Physiol. Nutr. Metab..

[4] McAnulty S.R., McAnulty L.S., Nieman D.C., Dumke C.L., Morrow J.D., Utter A.C., Henson D.A., Proulx W.R., George G.L. (2004). Consumption of blueberry polyphenols reduces exercise-induced oxidative stress compared to vitamin C. Nutr. Res..

[5] Trombold J.R., Reinfeld A.S., Casler J.R., Coyle E.F. (2011). The effect of pomegranate juice supplementation on strength and soreness after eccentric exercise. J. Strength Cond. Res..

[6] Cao G., Zuo J., Wu B., Wu Y. (2024). Polyphenol supplementation boosts aerobic endurance in athletes: Systematic review. Front. Physiol..

[7] Clauss M., Skattebo Ø., Rasen Dæhli M., Ditta Valsdottir T., Ezzatkhah Bastani N., Ivar Johansen E., Jensen Kolnes K., Steen Skålhegg B., Jensen J. (2023). Carbohydrate ingestion during prolonged cycling improves next-day time trial performance and alters amino acid concentrations. Med. Sci. Sports Exerc..

[8] Muros J.J., Sánchez-Muñoz C., Campos D., Hinojosa-Nogueira D., Rufián-Henares J.Á., Mateo-March M., Zabala M. (2022). Nutritional habits of professional cyclists during pre-season. Nutrients.

[9] García-Rovés P.M., Terrados N., Fernández S., Patterson A.M. (2000). Comparison of dietary intake and eating behavior of professional road cyclists during training and competition. Int. J. Sport Nutr. Exerc. Metab..

[10] Thomas D.T., Erdman K.A., Burke L.M. (2016). American College of Sports Medicine Joint Position Statement. Nutrition and Athletic Performance. Med. Sci. Sports Exerc..

[11] Ballester P., Cerdá B., Arcusa R., Marhuenda J., Yamedjeu K., Zafrilla P. (2022). Effect of ginger on inflammatory diseases. Molecules.

[12] Black C.D., O’Connor P.J. (2010). Acute effects of dietary ginger on muscle pain induced by eccentric exercise. Phytother. Res..

[13] Matsumura M.D., Zavorsky G.S., Smoliga J.M. (2015). The effects of pre-exercise ginger supplementation on muscle damage and delayed onset muscle soreness. Phytother. Res..

[14] Wilson P.B. (2020). A randomized double-blind trial of ginger root for reducing muscle soreness and improving physical performance recovery among experienced recreational distance runners. J. Diet. Suppl..

[15] Hattori S., Omi N., Yang Z., Nakamura M., Ikemoto M. (2021). Effect of ginger extract ingestion on skeletal muscle glycogen contents and endurance exercise in male rats. Phys. Act. Nutr..

[16] Ghayur M.N., Gilani A.H. (2005). Ginger lowers blood pressure through blockade of voltage-dependent calcium channels. J. Cardiovasc. Pharmacol..

[17] McKay A.K., Stellingwerff T., Smith E.S., Martin D.T., Mujika I., Goosey-Tolfrey V.L., Sheppard J., Burke L.M. (2022). Defining Training and Performance Caliber: A Participant Classification Framework. Int. J. Sports Physiol. Perform..

[18] Bowtell J., Kelly V. (2019). Fruit-derived polyphenol supplementation for athlete recovery and performance. Sports Med..

[19] Duracinsky M., Archbold S., Lobo B., Bessonneau P., Thonon F., Santos J., Guagnozzi D., Payakachat N., Coffin B., Azpiroz F. (2022). The Intestinal Gas Questionnaire (IGQ): Psychometric validation of a new instrument for measuring gas-related symptoms and their impact on daily life among general population and irritable bowel syndrome. Neurogastroenterol. Motil..

[20] Lichtenstein A.H., Petersen K., Barger K., Hansen K.E., Anderson C.A.M., Baer D.J., Lampe J.W., Rasmussen H., Matthan N.R. (2020). Perspective: Design and Conduct of Human Nutrition Randomized Controlled Trials. Adv. Nutr..

[21] Nieman D., Valacchi G., Wentz L., Ferrara F., Pecorelli A., Woodby B., Sakaguchi C., Simonson A. (2019). Mixed Flavonoid Supplementation Attenuates Postexercise Plasma Levels of 4-Hydroxynonenal and Protein Carbonyls in Endurance Athletes. Int. J. Sport Nutr. Exerc. Metab..

[22] Nieman D.C., Scherr J., Luo B., Meaney M.P., Dréau D., Sha W., Dew D.A., Henson D.A., Pappan K.L. (2014). Influence of Pistachios on Performance and Exercise-Induced Inflammation, Oxidative Stress, Immune Dysfunction, and Metabolite Shifts in Cyclists: A Randomized, Crossover Trial. PLoS ONE.

[23] Salgado R.M., Caldwell A.R., Coffman K.E., Cheuvront S.N., Kenefick R.W. (2020). Endurance test selection optimized via sample size predictions. J. Appl. Physiol..

[24] Stone M.R., Thomas K., Wilkinson M., Stevenson E., St Clair Gibson A., Jones A.M., Thompson K.G. (2017). Exploring the performance reserve: Effect of different magnitudes of power output deception on 4000 m cycling time-trial performance. PLoS ONE.

[25] Dantas J.L., Pereira G., Nakamura F.Y. (2015). Five-kilometers time trial: Preliminary validation of a short test for cycling performance evaluation. Asian J. Sports Med..

[26] MacRae H.S., Mefferd K.M. (2006). Dietary antioxidant supplementation combined with quercetin improves cycling time trial performance. Int. J. Sport Nutr. Exerc. Metab..

[27] Cook M.D., Myers S.D., Blacker S.D., Willems M.E.T. (2015). New Zealand blackcurrant extract improves cycling performance and fat oxidation in cyclists. Eur. J. Appl. Physiol..

[28] Roberts J.D., Roberts M.G., Tarpey M.D., Weekes J.C., Thomas C.H. (2015). The effect of a decaffeinated green tea extract formula on fat oxidation, body composition and exercise performance. J. Int. Soc. Sports Nutr..

[29] Hooper D.R., Orange T., Gruber M.T., Darakjian A.A., Conway K.L., Hausenblas H.A. (2021). Broad spectrum polyphenol supplementation from tart cherry extract on markers of recovery from intense resistance exercise. J. Int. Soc. Sports Nutr..

[30] Trinity J.D., Pahnke M.D., Trombold J.R., Coyle E.F. (2014). Impact of polyphenol antioxidants on cycling performance and cardiovascular function. Nutrients.

[31] Black C.D., Oconnor P.J. (2008). Acute effects of dietary ginger on quadriceps muscle pain during moderate-intensity cycling exercise. Int. J. Sport Nutr. Exerc. Metab..

[32] Demirli A., Ulupınar S., Terzi M., Özbay S., Özkara A.B., Gençoğlu C., Ouergui I., Ardigò L.P. (2025). Synergistic Effects of Green Tea Extract and Ginger Supplementation on Endurance Performance and Thermal Perception in Normothermic and Cold Environments: A Randomized, Placebo-Controlled, Double-Blind Crossover Trial. Nutrients.

[33] Sanchez Diaz M., Martin-Castellanos A., Fernandez-Elias V.E., Lopez Torres O., Lorenzo Calvo J. (2022). Effects of polyphenol consumption on recovery in team sport athletes of both sexes: A systematic review. Nutrients.

[34] Ferrara L., D’Angelo S. (2023). Post-exercise fatigue, lactate, and natural nutritional strategy. J. Phys. Educ. Sport.

[35] Arrese A.L., Ostáriz E.S., Mallen J.C., Izquierdo D.M. (2005). The changes in running performance and maximal oxygen uptake after long-term training in elite athletes. J. Sports Med. Phys. Fit..

[36] Tanaka H., Seals D.R. (2008). Endurance exercise performance in Masters athletes: Age-associated changes and underlying physiological mechanisms. J. Physiol..

[37] Lepers R., Stapley P.J. (2016). Master Athletes Are Extending the Limits of Human Endurance. Front. Physiol..

[38] Fleg J.L., Morrell C.H., Bos A.G., Brant L.J., Talbot L.A., Wright J.G., Lakatta E.G. (2005). Accelerated Longitudinal Decline of Aerobic Capacity in Healthy Older Adults. Circulation.

[39] Christou D.D., Seals D.R. (2008). Decreased maximal heart rate with aging is related to reduced β-adrenergic responsiveness but is largely explained by a reduction in intrinsic heart rate. J. Appl. Physiol..

[40] Coyle E.F. (1999). Physiological determinants of endurance exercise performance. J. Sci. Med. Sport.

[41] Matusiak-Wieczorek E., Pyciarz L., Drobniewski M., Borowski A. (2023). An assessment of the dietary habits among road cyclists competing in amateur races. Food Sci. Nutr..

[42] Stearns R.L., Emmanuel H., Volek J.S., Casa D.J. (2010). Effects of ingesting protein in combination with carbohydrate during exercise on endurance performance: A systematic review with meta-analysis. J. Strength Cond. Res..

[43] Kinney A.R., Eakman A.M., Graham J.E. (2020). Novel effect size interpretation guidelines and an evaluation of statistical power in rehabilitation research. Arch. Phys. Med. Rehabil..

[44] Kluboito Y., Mintah J.K., Essien-Baidoo S., Arthur N.A. (2024). Effect of acute supplementation of hibiscus-ginger drink on university athletes’ aerobic power and blood lactate. N. Afr. J. Food Nutr. Res..

[45] Pérez D.I.V., Mozer R.L., dos Santos D.A., Silva E.F., Queiroz A.C.C., Miarka B., Brito C.J., Quintana M.S. (2020). Suplementação aguda de substrato de gengibre não aumenta o metabolismo em repouso e durante o exercício. Motricidade.

[46] Zehsaz F., Farhangi N., Mirheidari L. (2014). Clinical immunology The effect of Zingiber officinale R. rhizomes (ginger) on plasma pro-inflammatory cytokine levels in well-trained male endurance runners. Cent. Eur. J. Immunol..

